# Deciphering Drought Resilience in Solanaceae Crops: Unraveling Molecular and Genetic Mechanisms

**DOI:** 10.3390/biology13121076

**Published:** 2024-12-20

**Authors:** Xin Pang, Jun Chen, Linzhi Li, Wenjuan Huang, Jia Liu

**Affiliations:** 1 Suzhou Polytechnic Institute of Agriculture, Suzhou 215008, China; pangx@szai.edu.cn (X.P.); chenjun@szai.edu.cn (J.C.); llli@szai.edu.cn (L.L.); 2 Wulanchabu Academy of Agricultural and Forestry Sciences, Wulanchabu 012000, China; huangwenjuan_013@163.com; 3 College of Agriculture, Inner Mongolia Agricultural University, Hohhot 010018, China

**Keywords:** Solanaceae crops, drought-resistant, research progress

## Abstract

The Solanaceae family, which includes vital crops such as tomatoes, peppers, eggplants, and potatoes, is increasingly impacted by drought due to climate change. Recent research has concentrated on unraveling the molecular mechanisms behind drought resistance in these crops, with a focus on abscisic acid (ABA) signaling pathways, transcription factors (TFs) like MYB (Myeloblastosis), WRKY (WRKY DNA-binding protein), and NAC (NAM, ATAF1/2, and CUC2- NAM: No Apical Meristem, ATAF1/2, and CUC2: Cup-shaped Cotyledon), and the omics approaches. Moreover, transcriptome sequencing (RNA-seq) has been instrumental in identifying differentially expressed genes (DEGs) crucial for drought adaptation. Proteomics studies further reveal changes in protein expression under drought conditions, elucidating stress response mechanisms. Additionally, microRNAs (miRNAs) have been identified as key regulators in drought response. Advances in proteomics and transcriptomics have highlighted key proteins and genes that respond to drought stress, offering new insights into drought tolerance. To address the challenge of drought, future research should emphasize the development of drought-resistant varieties through precision breeding techniques such as gene editing, marker-assisted selection (MAS), and the integration of artificial intelligence. Additionally, the adoption of environmentally sustainable cultivation practices, including precision irrigation and the use of anti-drought agents, is crucial for improving water-use efficiency and crop resilience. International collaboration and data sharing will be essential to accelerate progress and ensure global food security in increasingly arid conditions. These efforts will enable Solanaceae crops to adapt the challenges posed by climate change, ensuring their productivity and sustainability.

## Simple Summary

This study addresses the challenge of drought stress, which significantly threatens crops in the Solanaceae family, such as tomatoes, peppers, eggplants, and potatoes. Drought stress disrupts plant growth, reduces yields, and impacts food security. Our research focuses on understanding how these plants respond to drought at the molecular level. In this review, key genes, proteins, and microRNAs—small molecules that regulate Solanaceae plant responses—linked to drought tolerance were summarized. By analyzing gene expression and protein changes in stressed plants, we identified essential components that help plants conserve water, maintain energy balance, and protect against damage. We also highlight the importance of breeding strategies to enhance drought tolerance in these crops, using both traditional methods and advanced technologies like gene editing. This research provides valuable insights for developing hardier, drought-resistant crops. Ultimately, these findings could lead to better agricultural practices and more resilient crops, helping to secure food production in the face of climate change and water scarcity, benefiting both farmers and society.

## 1. Introduction

The Solanaceae family is a large family that includes over 70 genera and more than 2000 species, with about 16 genera and 70 species found in China. This family includes many important vegetable crops such as tomato (*Solanum lycopersicum*), pepper (*Capsicum annuum*), eggplant (*Solanum melongena*), and potato (*Solanum tuberosum*). According to data from the Food and Agriculture Organization of the United Nations (FAO) in 2022, the planting area of these four Solanaceae vegetable crops in China reached 7.704 million hectares, with a total production of 202.326 million tons, making them a significant part of China’s vegetable industry.

Drought stress detrimentally affects the vegetative growth of Solanaceae species, leading to reduced plant height, leaf surface area, and photosynthetic efficiency, ultimately resulting in lower crop yield and diminished quality. Current research on drought resistance in Solanaceae crops predominantly explores alterations in growth patterns, physiological and biochemical responses, and photosynthetic activity. However, the exploration of the genetic loci and underlying molecular mechanisms governing drought resistance remains relatively underdeveloped. This review aims to systematically summarize the research progress in recent years on the effects of drought on Solanaceae plants, the molecular mechanisms, and molecular biology studies. It will provide a theoretical basis for basic research on drought resistance in Solanaceae plants and the breeding of new drought-resistant varieties.

The mechanisms of plant drought resistance can be classified into three main types: drought avoidance, drought escape, and drought tolerance [1]. This review primarily focuses on the molecular and genetic mechanisms of drought tolerance in Solanaceae crops, given the extensive body of research available on this topic. Although strategies like drought avoidance (e.g., stomatal regulation and root architecture modification) and drought escape (e.g., early flowering) are crucial, detailed molecular insights specific to these mechanisms in Solanaceae crops remain relatively scarce. Recent advances in understanding drought tolerance in Solanaceae plants have significantly enriched our knowledge of their molecular, physiological, and biochemical adaptations. Molecular studies have identified key transcription factors, including MYB, WRKY, and NAC, which play pivotal roles in regulating drought-responsive gene expression and facilitating stress adaptation [2]. On the physiological front, research has underscored the importance of ABA signaling pathways in modulating plant responses to water scarcity, with both ABA-dependent and ABA-independent mechanisms contributing to enhanced drought resilience [3]. From a biochemical perspective, notable progress includes the identification of critical proteins and enzymes, such as antioxidant enzymes and osmoprotectants, which help mitigate oxidative damage and preserve cellular functionality under stress conditions [4]. While significant strides have been made in identifying molecules associated with drought responses in Solanaceae crops, unraveling their specific functional roles and their integration into complex regulatory networks remains a critical challenge. This review aims to provide a comprehensive overview of the abundance of drought-responsive molecules while offering insights into their specific roles and interactions within molecular pathways.

During plant growth, various abiotic stresses are encountered, among which drought is one of the most destructive factors, severely affecting crop yield and quality. According to the “2023 Global Drought Report” released by the United Nations, 15–20% of China’s population faces more frequent moderate to severe droughts in this century, and by 2100, the intensity of drought in China is expected to increase by 80%. To address the increasingly severe drought conditions and ensure the demand for crop yield and food security in human society, accelerating research on crop drought resistance has become particularly important.

## 2. Plant Drought-Resistance Mechanisms

### 2.1. Drought Avoidance

Drought avoidance (DA) refers to the ability of plants to avoid the negative effects of mild drought stress by regulating certain morphological structures or growth rates, thereby maintaining a certain water status and normal physiological functions under drought conditions. The key characteristic of drought avoidance is the regulation of plant water potential through changes in leaves and roots under water-deficient conditions [5]. On one hand, plants control transpiration by regulating stomatal movement to reduce water loss; on the other hand, they alter the structure, number, and length of roots to absorb more water [6]. Drought avoidance in Solanaceae crops primarily involves stomatal regulation and root system adjustments. Studies have shown that ABA signaling plays a pivotal role in controlling stomatal closure to reduce water loss. Genes such as *SlAREB1* (*Solanum lycopersicum* abscisic acid-responsive element binding protein 1) in tomatoes [7] and *CaAIMK1* (*Capsicum annuum* ABA-induced MAP Kinase 1) [8] in peppers have been implicated in this process, enhancing water-use efficiency under drought conditions. Additionally, deeper and more extensive root systems, regulated by key root development genes, contribute to drought avoidance, although specific studies in Solanaceae remain limited.

### 2.2. Drought Escape

Drought escape (DE) is the strategy where plants adjust their life cycle by flowering earlier or later to avoid drought, completing their entire developmental cycle in a shorter time [1]. Both genotype and environment determine the growth and development cycle of plants, as well as their ability to escape from abiotic stress. When plants encounter drought or other adverse conditions during their growing season, the timing of flowering and seed maturation is a key indicator of their adaptability to the environment. Therefore, breeding early maturing varieties is one of the effective ways for plants to avoid stress from drought and other adverse conditions, thereby increasing crop yield. Drought escape involves completing the life cycle before the onset of severe water stress, often through early flowering. In Solanaceae crops, genes controlling flowering time (FT), such as FT-like genes, have been identified as potential regulators of this mechanism [9]. While this strategy has been studied extensively in other crops like rice, targeted research on Solanaceae remains sparse. Breeding programs aimed at early maturing varieties could offer promising avenues for drought escape in these crops.

### 2.3. Drought Tolerance

Drought tolerance (DT) refers to the ability of plants to enhance their resistance to drought through physiological and biochemical regulatory substances produced by their genetic mechanisms [1]. The enhancement of drought tolerance in plants primarily involves two types. The first is the production and secretion of osmotic regulatory substances to increase water absorption and maintain cell osmotic potential. Osmotic regulatory substances include proline, organic acids, glycine, potassium, soluble sugars, sugar alcohols, betaine, chloride ions, among others [10,11]. The second type is to enhance the antioxidant capacity of cells, improving the ability to eliminate reactive oxygen species. The plant antioxidant system consists of enzymatic and non-enzymatic systems. The enzymatic system includes catalase, peroxidase, superoxide dismutase, etc.; the non-enzymatic system includes ascorbic acid, reduced glutathione, and cysteine [12]. The increase in enzyme activity in the enzymatic system and the content of substances in the non-enzymatic system contribute to improving plant drought tolerance.

## 3. Molecular Mechanisms of Drought Resistance in Solanaceae Plants

**Figure 1:**
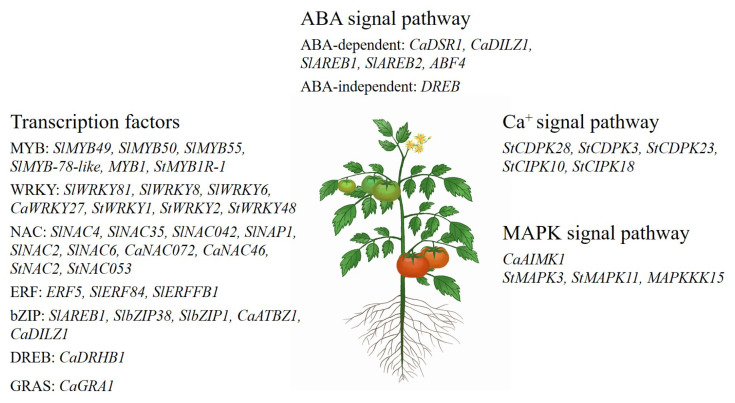
List of genes and gene families of Solanaceae crops response to drought stress.

Solanaceae crops have evolved various molecular mechanisms that respond to drought stress through changes in gene expression and metabolic pathways (Figure 1).

### 3.1. Role of Abscisic Acid Pathways in Regulating Drought Stress Responses in Solanaceae Crops

Abscisic acid is one of the natural plant growth regulators involved in plant growth and development, fruit development, flowering regulation, and responses to abiotic stresses such as drought and salinity. ABA can reduce transpiration rate and water loss by promoting stomatal closure, sense and transmit drought stress signals as signaling molecules, and regulate the expression of drought stress-related genes to help plants adapt to drought environments [13]. Drought-responsive gene expression, mediated by abscisic acid, involves both ABA-dependent and ABA-independent signaling pathways, each contributing to enhanced water stress resilience [14]. For example, the AREB/ABF (abscisic acid-responsive element binding protein/abscisic acid-responsive element binding factor) family is an ABA-dependent pathway and the DREB/CBF (Dehydration-responsive element binding/C-repeat Binding factor) family is the ABA-independent pathway. Lim et al. identified two key genes in pepper that are involved in ABA-mediated drought stress signaling: *CaDSR1* (*Capsicum annuum* Drought Sensitive RING finger protein 1) and *CaDILZ1* (*Capsicum annuum* Drought-Induced Leucine Zipper 1) [15]. These genes encode proteins that interact with ABA signaling components, modulating the plant’s response to water deficit. *CaDSR1* has been shown to enhance the expression of stress-responsive genes, while *CaDILZ1* appears to be involved in the regulation of ABA levels and sensitivity [15]. These findings underscore the complex interplay between ABA signaling and gene expression in the context of drought tolerance.

The expression of *SlAREB1* and *SlAREB2* in tomato roots and stems was induced by drought stress. *SlAREB1*-overexpressing transgenic tomato plants showed increased tolerance to water stress compared to wild-type and *SlAREB1*-downregulating transgenic plant. DNA microarray analysis of gene expression showed that *SlAREB1* was involved in the regulation of genes related to oxidative stress and ABA signaling pathway [7]. Key transcription factors such as *SlAREB1* and *SlAREB2* have been identified as regulators of the ABA-dependent pathway in tomatoes, enhancing drought tolerance by upregulating oxidative stress-related genes and ABA-responsive genes. Functional studies have shown that overexpression of *SlAREB1* significantly improves drought tolerance, while its silencing reduces stress resilience, emphasizing its critical role in ABA signaling [7].

In potatoes, research has demonstrated the effectiveness of manipulating both ABA-dependent and ABA-independent pathways to enhance drought tolerance. Watanabe et al. conducted a study on transgenic potatoes expressing the *AtDREB1a* gene, which is part of the DREB/CBF family and functions through the ABA-independent pathway [16]. The transgenic lines exhibited significantly enhanced drought tolerance compared to non-transgenic controls. This enhancement was attributed to the upregulation of stress-responsive genes that improve osmotic adjustment and protect cellular structures during water deficit conditions [16]. Jia et al. also demonstrated that overexpression of Arabidopsis *DREB1A* resulted in improved drought stress tolerance in *S. tuberosum* plants [17].

Another study by Muñiz García et al. focused on the overexpression of the *AtABF4* gene in potatoes, which is part of the AREB/ABF family and operates through the ABA-dependent pathway [18]. The overexpression of *AtABF4* led to improved drought tolerance, as evidenced by higher tuber yield under drought conditions. This improvement was linked to the activation of ABA-responsive genes that enhance water-use efficiency and reduce oxidative stress. The results of this study highlight the potential of manipulating ABA-dependent pathways to improve drought resistance in crops [18]. Yang et al. found that one ABF TF gene was significantly upregulated under polyethylene glycol (PEG)-induced drought stress, and this TF could bind to the downstream *cis*-acting regulatory element ABRE and regulate the expression of ABA response genes [19].

### 3.2. Transcription Factor Networks in Solanaceae: Regulating Drought Resistance Through MYB, WRKY, and NAC Families

Drought stress triggers a complex network of signaling pathways that activate the expression of genes involved in water retention, osmotic adjustment, and protection against oxidative damage. Transcription factors play a pivotal role in drought stress signaling networks by binding to specific promoter regions of target genes, modulating transcriptional activity to orchestrate adaptive physiological and molecular responses. In Solanaceae plants, several transcription factor families have been identified as key regulators of drought stress responses, including MYB, WRKY, ERF (Ethylene Response Factor), NAC, and bZIP (basic region/leucine zipper). The roles of transcription factors in drought resistance are summarized in Table 1. Genes that enhance drought tolerance are categorized as ‘positive regulators’, while those that suppress adaptive responses are listed as ‘negative regulators’. The revised terminology reflects the functional impact of each gene on stress resistance, providing a clearer understanding of their roles in drought adaptation (Table 1).

#### 3.2.1. MYB Transcription Factors

The MYB family is one of the largest and most diverse transcription factor families in plants. MYB proteins are involved in various physiological processes, including responses to abiotic stress. In tomatoes, MYB transcription factors have been shown to play significant roles in regulating drought resistance. For instance, several MYB genes have been identified to positively regulate drought tolerance by modulating the expression of genes involved in osmotic regulation and antioxidant defense [20,21]. In peppers and potatoes, MYB transcription factors also contribute to drought resistance by regulating similar stress-responsive pathways [39,46]. For example, Chen et al. pointed out that the MYB family transcription factor *SlMYB55* is the ABA and drought response gene, and silencing and expressing *SlMYB55* can significantly improve the drought resistance of tomato. In addition, *SlMYB55* can regulate the synthesis and signaling pathway of ABA [21].

#### 3.2.2. WRKY Transcription Factors

WRKY transcription factors are another important family involved in plant stress responses. These proteins are characterized by the WRKY domain, which binds to W-box elements in the promoters of target genes. WRKY transcription factors have been shown to regulate a wide range of stress-responsive genes, particularly those involved in hormone signaling and reactive oxygen species (ROS) scavenging. In peppers and potatoes, WRKY transcription factors also play crucial roles in enhancing drought resistance by modulating stress-responsive gene expression [47,48,49]. Ahammed et al. found that *SlWRKY81* could reduce proline synthesis and reduce tomato tolerance to drought. The expression of *SlWRKY81* was upregulated under drought conditions. After *SlWRKY81* was silenced, stomatal closure of tomato was accelerated under drought stress, and drought-induced damage was significantly reduced. Overexpression of *SlWRKY8* can accelerate stomatal closure, promote the expression of stress response genes *SlAREB*, *SlDREB2A* and *SlRD29*, increase the accumulation of proline, reduce the accumulation of malondialdehyde (MDA) and hydrogen peroxide (H_2_O_2_), and thus improve the drought resistance of tomato [23,24].

#### 3.2.3. ERF Transcription Factors

ERF family is part of the larger AP2/ERF superfamily, which is known for its involvement in various stress responses, including drought. ERF transcription factors regulate the expression of genes involved in ethylene signaling, which plays a crucial role in the plant’s response to abiotic stress. In tomatoes, ERF transcription factors have been shown to modulate drought tolerance by regulating genes involved in cell wall modification, osmotic regulation, and ROS scavenging [27,28,29]. The role of ERF transcription factors in peppers and potatoes is less well-documented, but they are believed to contribute to drought resistance through similar mechanisms.

#### 3.2.4. NAC Transcription Factors

NAC transcription factors are widely recognized for their roles in regulating plant development and stress responses. In the context of drought stress, NAC transcription factors regulate genes involved in water retention, osmotic adjustment, and detoxification of ROS. In tomatoes, several NAC genes have been identified to positively regulate drought tolerance. These genes are involved in modulating the expression of key drought-responsive genes, including those related to ABA signaling and osmotic regulation [31,32,33,34,35]. In peppers and potatoes, NAC transcription factors have also been shown to enhance drought resistance by activating similar stress-responsive pathways [41,42,50,51]. The cloning of a potato NAC transcription factor gene *StNAC053*, which was significantly upregulated after drought and abscisic acid treatments. *StNAC053* may function as a transcriptional activator in potato. *Arabidopsis* plants overexpressing *StNAC053* displayed lower seed germination rates compared to wild-type under exogenous ABA treatment. In addition, the *StNAC053* overexpression Arabidopsis lines displayed significantly increased tolerance to drought stress treatments. Moreover, the *StNAC053-OE* lines were found to have higher activities of catalase (CAT), superoxide dismutase (SOD) and peroxidase (POD) under drought treatments [51].

#### 3.2.5. bZIP Transcription Factors

The basic leucine zipper family of transcription factors is involved in various plant stress responses, including drought. bZIP proteins regulate the expression of genes involved in ABA signaling, which is a critical pathway for drought tolerance. In tomatoes, bZIP transcription factors have been shown to enhance drought resistance by activating the expression of ABA-responsive genes that help maintain water balance and protect against oxidative damage [7,36,37]. Although it is less studied in peppers and potatoes, bZIP transcription factors are likely to play similar roles in these crops, contributing to their drought resistance [15].

### 3.3. Kinase Signaling Pathways in Solanaceae: Enhancing Drought Resistance Through MAPK and CDPK Networks

Research has shown that, in addition to the abscisic acid signaling pathway, certain kinase signaling pathways like mitogenactivated protein kinase (MAPK), Calcium-dependent protein kinases (CDPK) and CBL-interacting protein kinase (CIPK) are also involved in Solanaceae plants’ response to drought stress. In the pepper genome, 27 MAPKKK gene families have been identified, with nine of these showing transcriptional regulation in response to ABA and drought stress [8]. This indicates a significant overlap between the MAPK and ABA signaling pathways, suggesting that MAPKKKs may act as key nodes integrating signals from different pathways to fine-tune the drought response. The isolated *CaAIMK1*, a MAPK kinase, has been demonstrated to enhance drought tolerance in peppers through the ABA-dependent pathway. This kinase likely activates downstream MAPKs that regulate the expression of stress-responsive genes, contributing to improved water-use efficiency and osmotic balance under drought conditions. The photosynthesis and stomatal conductance of potatoes treated with PEG and mannitol are regulated by *StMAPK3*, which indicates that *StMAPK3* is a regulator of drought/osmotic stress [52]. Analysis of the expression levels of 15 potato MAPKs showed that *StMAPK11* was significantly upregulated under drought conditions, and it enhanced the drought resistance of potato plants through antioxidant activity and photosynthesis [53]. RNA-seq analysis of drought-tolerant and drought-sensitive varieties identified 22 drought response genes in potato, including mitogen-activated protein kinase kinase 15 (MAPKKK15), which has been shown to be activated at the transcriptional level under drought conditions [54].

Under drought and osmotic stress conditions, overexpression of *StCDPK28* in potatoes has been shown to significantly enhance drought tolerance. This improvement is associated with a reduction in MDA and H_2_O_2_ content, which are indicators of lipid peroxidation and oxidative stress, respectively. In addition, the overexpression of *StCDPK28* also leads to increased activities of antioxidant enzymes such as CAT, superoxide SOD, and POD, which help mitigate oxidative damage by scavenging ROS. These findings suggest that *StCDPK28* enhances the plant’s antioxidant defense system, thereby reducing cellular damage under drought stress [55]. Bi et al. also found *StCDPK3* and *StCDPK23* genes were significantly upregulated under drought stress [56]. Overall, The MAPK signaling pathway plays a critical role in drought adaptation. For example, *StMAPK3* in potatoes regulates photosynthesis and stomatal conductance under osmotic stress [52], while *StCDPK28* enhances antioxidant defenses by increasing catalase and superoxide dismutase activities, reducing oxidative damage [56].

In potatoes, *StCIPK10* has been identified as a key regulator of drought and osmotic stress tolerance. Overexpression of *StCIPK10* enhances the plant’s ability to scavenge ROS and increases the accumulation of osmotic regulatory substances, such as proline and soluble sugars, which help maintain cellular osmotic balance under water deficit conditions [57]. Similarly, *StCIPK18* has been shown to be upregulated under drought stress, and its overexpression leads to improved water retention capacity in potato plants. This is evidenced by a reduction in leaf water loss rate and MDA content, alongside increased proline content and the activities of CAT, SOD, and POD [58].

The identification of transcription factors, kinases, and proteomic changes under drought stress has provided critical insights into the molecular mechanisms of drought tolerance. Functional validation studies, such as overexpression of *SlWRKY8* in tomatoes, have demonstrated its role in modulating stomatal closure and oxidative stress [25]. Similarly, *StCDPK28* in potatoes enhances the antioxidant defense system, highlighting the interconnected roles of these molecules in mitigating drought-induced damage [56]. These findings underscore the importance of linking molecular abundance with specific functional roles to develop targeted strategies for improving drought resilience in Solanaceae crops.

### 3.4. Role of Osmoprotective Metabolites in Drought Tolerance

Osmoprotective metabolites, including soluble sugars and proline, play a critical role in biochemical adaptation to drought stress. Soluble sugars, such as sucrose and trehalose, are essential for maintaining osmotic balance, stabilizing cellular structures, and protecting membranes and proteins from dehydration-induced damage [11]. In Solanaceae crops, studies have reported an increase in sugar accumulation during drought stress, contributing to improved water retention and metabolic stability [59].

Proline, another key osmoprotectant, serves dual functions in drought tolerance. It acts as an osmolyte to maintain cell turgor and as a scavenger of ROS, mitigating oxidative damage [60]. For example, drought-tolerant tomato genotypes exhibit elevated proline levels, correlating with enhanced stress resilience [61]. In potatoes, similar findings highlight the role of proline in maintaining cellular integrity under water deficit conditions [62].

These metabolites are regulated by transcription factors such as MYB and NAC, which activate their biosynthesis pathways under drought conditions. For instance, genes involved in proline biosynthesis, such as P5CS (Δ1-pyrroline-5-carboxylate synthetase), are upregulated in response to drought stress in tomatoes and potatoes, further underscoring their importance in drought resilience [63].

## 4. Omics Approaches in Drought Tolerance Research in Solanaceae Plants

### 4.1. Application of Transcriptome Sequencing in Drought Stress of Solanaceae Plants

Transcriptome sequencing technology (RNA-seq) is a powerful tool that allows for the high-throughput sequencing of RNA transcripts, providing a global snapshot of gene expression profiles under specific conditions. In the context of drought stress, RNA-seq has been instrumental in identifying differentially expressed genes (DEGs) that play crucial roles in the plant’s adaptive responses. These DEGs are involved in various biological processes, including hormone signaling, metabolic pathways, and stress response mechanisms.

Nicolas et al. profiled the transcriptomes of a spectrum of fruit tissues from tomato (*Solanum lycopersicum*), and the results highlight the spatiotemporal specificity of drought responses in tomato fruit and indicate known and unrevealed molecular regulatory mechanisms involved in drought acclimation, during both vegetative and reproductive stages of development [64]. Shu et al. analyzed the global transcriptional and metabolic changes in *S. pimpinellifolium* and *S. lycopersicum* under drought and recovery conditions, 38 genes involved in metabolic pathways, the biosynthesis of secondary metabolites, the biosynthesis of amino acids, and ABC transporters related to responses to water stress [65].

Sprenger et al. conducted a transcriptome sequencing analysis on drought-tolerant and drought-sensitive potato varieties under both greenhouse and field conditions. Their study revealed that 54 genes were consistently upregulated, while 48 genes were consistently downregulated under drought stress. Notably, the upregulated genes included those encoding heat shock proteins (HSP20, HSP70), the abscisic acid receptor PYL4, and several kinases such as MAPK kinase, phosphorylated extracellular signal-regulated kinase 1 (GWAS 1 kinase), and receptor kinase. These findings highlight the activation of stress-responsive pathways that contribute to the enhanced drought tolerance observed in certain potato varieties [66]. Yang et al. used P3198, a diploid potato genotype with strong drought resistance, as experimental material to study the drought response genes under PEG-induced stress by RNA sequencing technology. A total of 1665 DEGs were identified, and annotations showed that these DEGs primarily encode transcription factors, protein kinases, and proteins associated with redox regulation, carbohydrate metabolism, and osmoregulation [19]. The drought-tolerant potato landrace was treated with drought stress, rehydration and re-dehydration, and RNA-seq was applied to analyze the characteristics of gene regulation during these treatments. The results showed that drought-responsive genes mainly involved photosynthesis, signal transduction, lipid metabolism, sugar metabolism, wax synthesis, cell wall regulation, osmotic adjustment [67].

### 4.2. Application of Proteomics Sequencing in Drought Stress of Solanaceae Plants

With the publication of whole genome sequences and advancements in protein separation and quantification techniques, proteomics research under stress conditions has increased in major crops in recent years. In Solanaceae plants, drought stress research using proteomics has been conducted primarily on tomato and potato.

Marjanović et al. studied the effects of partial rootzone drying (PRD) on tomato fruit growth and fruit skin proteomics. They extracted proteins from fruit skin tissue at two stages of fruit growth (15 and 30 days after flowering) and conducted proteomic analysis. The expression of cell wall, energy-related, and stress defense-related proteins increased in PRD fruits, potentially extending the growth period of PRD fruits. The upregulation of some antioxidant enzymes during the cell expansion phase of PRD fruits might be related to their protection against mild stress induced by PRD [68]. Zhou et al. conducted proteomic analysis on the roots of a sensitive tomato variety (LA3465) and a dehydration-tolerant tomato variety (LA1958). They identified 130 proteins in LA3465, with 104 being suppressed and 26 induced, while 170 proteins were identified in LA1958, with 106 being suppressed and 64 induced. The changes in protein abundance caused by dehydration treatment indicated that stress inhibited cellular metabolic activity and protein biosynthesis [69]. Tamburino et al. studied the proteomics of tomato chloroplasts under severe drought treatment and recovery periods, revealing that water deficit significantly affected the chloroplast proteome (31 differentially expressed components), mainly involving energy-related functional proteins. The proteomics of the recovery phase showed a broader range of chloroplast proteins compared to the drought period (54 differentially expressed components). The changes in candidate gene expression and ABA accumulation indicated that stress activated specific retrograde signaling pathways from chloroplasts to the nucleus, interconnected with the ABA-dependent network [70]. Ogden et al. collected phloem exudates from tomato during drought stress and recovery, identifying a total of 2558 proteins, of which 169 showed significant changes in abundance. Proteins that significantly increased during drought included those involved in lipid metabolism, chaperone-mediated protein folding, carboxylic acid metabolism, ABA signaling, cytokinin biosynthesis, and amino acid metabolism [71].

Boguszewska-Mańkowska et al. compared two potato cultivars with different dehydration tolerances. Liquid chromatography–tandem mass spectrometry (LC–MS/MS) protein identification revealed that in the roots of the sensitive cultivar, drought stress led to an increased abundance of defense- and detoxification-related proteins. In contrast, the tolerant cultivar exhibited significant changes in the abundance of proteins associated with energy and carbohydrate metabolism [62]. Proteomics studies have identified heat shock proteins (e.g., HSP70) and antioxidant enzymes (e.g., catalase, superoxide dismutase) as pivotal players in protecting cellular structures under drought conditions [62]. In potatoes, the upregulation of HSP70 correlates with enhanced cellular protection, while increased catalase activity mitigates oxidative stress during drought [61].

### 4.3. Role of miRNA in Drought Stress of Solanaceae Plants

MicroRNAs (miRNAs), a class of small non-coding RNA molecules, play a pivotal role in drought stress response by fine-tuning gene expression at both transcriptional and translational levels. Among the miRNAs identified, miR169 has garnered attention for its role as a positive regulator under drought conditions in tomato. Zhang et al. demonstrated that miR169 expression is upregulated in response to drought, leading to the downregulation of its target gene, which is involved in the regulation of stress responses. This finding highlights miR169 as a critical component of the tomato’s drought response mechanism, potentially offering a target for genetic manipulation to enhance drought tolerance [72].

Further expanding on this, Candar-Cakir et al. subjected two different tomato varieties to drought stress and conducted a comprehensive analysis of miRNA expression. Their study identified 10 miRNAs that were differentially expressed in response to drought, indicating that multiple miRNAs are involved in the complex regulatory networks that govern drought tolerance in tomatoes. The identification of these miRNAs provides valuable insights into the genetic basis of drought resistance, and their functional characterization could lead to the development of drought-resistant tomato varieties through biotechnological approaches [73].

Liu et al. provided additional insights into the role of miRNAs in drought stress by analyzing the miRNA profiles of the tomato cultivar M82 and its introgression line IL25 under drought conditions. Their study revealed a total of 100 differentially expressed miRNAs, including 32 conserved miRNAs and 68 novel miRNAs. The differential expression patterns observed suggest that both conserved and novel miRNAs play crucial roles in modulating the tomato’s response to drought stress. Notably, the conserved miRNAs may regulate essential and evolutionarily conserved drought-responsive pathways, while novel miRNAs could be involved in species-specific adaptations to drought conditions [74].

Studies have also shown that miRNA families such as miR159, miR172, miR811, miR814, miR835, and miR4398 respond to drought stress in potato [75,76,77], while miR2673 and miR6461 may regulate the Pyrroline-5-carboxylate reductase (P5CR) and Proline dehydrogenase (ProDH) genes, respectively, in response to drought stress [78].

## 5. Conclusions

Drought stress poses a significant threat to the productivity and sustainability of Solanaceae crops such as tomatoes, peppers, eggplants, and potatoes. This review highlights key advancements in understanding the molecular and genetic mechanisms of drought tolerance, including the roles of ABA signaling, transcription factors like MYB, WRKY, and NAC, and small regulatory molecules like microRNAs. Omics studies have identified important genes and proteins, such as *SlAREB1* and HSP70, that contribute to water conservation, osmotic adjustment, and oxidative stress protection.

While drought tolerance mechanisms are well-studied, strategies like drought avoidance (e.g., stomatal regulation) and drought escape (e.g., early flowering) need further investigation in Solanaceae crops. These approaches, combined with advanced breeding techniques and sustainable farming practices, hold potential for improving drought resilience. Moving forward, integrating multi-omics data and focusing on the functional roles of key molecules will help develop new drought-resistant varieties. These efforts are critical for adapting Solanaceae crops to climate challenges and ensuring global food security.

## 6. Future Perspectives

The Solanaceae family, which includes crops like tomatoes, peppers, eggplants, and potatoes, plays a crucial role in global agriculture. However, as climate change intensifies, drought has emerged as a significant threat to the yield and quality of these crops. To address this challenge, future research and development in Solanaceae drought resistance will likely focus on several key areas.

Firstly, a deeper understanding of the molecular mechanisms underlying drought resistance is essential. Current research has primarily focused on ABA signaling pathways, transcription factors, and the functional identification of some drought-resistant genes. However, a comprehensive regulatory network for drought resistance has yet to be fully developed. Future efforts should leverage modern molecular biology techniques such as gene editing, genome-wide association studies (GWAS), and transcriptome sequencing to uncover the gene expression, signal transduction, and metabolic regulation networks in Solanaceae plants under drought stress. Integrating multi-omics data to construct a regulatory network and interaction map of drought resistance will provide a solid theoretical foundation for breeding drought-resistant varieties.

Secondly, the development of new drought-resistant varieties will be a top priority. While traditional breeding methods have made significant progress in improving crop yield and disease resistance, they have proven inadequate in addressing extreme climate conditions. Future breeding efforts should focus on identifying and utilizing naturally drought-resistant germplasm resources. Techniques such as marker-assisted selection and gene editing can be used to precisely locate and modify key drought-resistance genes. Additionally, integrating artificial intelligence and big data technologies into the breeding process can optimize breeding strategies, shorten breeding cycles, and enhance efficiency. These precision breeding strategies have the potential to produce new Solanaceae varieties with enhanced drought tolerance and adaptability.

Moreover, the promotion of environmentally friendly cultivation techniques will be crucial. Alongside improving the drought resistance of Solanaceae plants, effective water resource management and optimized cultivation practices will play vital roles. For instance, the adoption of precision irrigation, mulching, and the use of anti-drought agents can improve water-use efficiency, enhance plant drought tolerance, and ensure stable crop yields under drought conditions.

Lastly, international collaboration and data sharing will be critical for future progress. Drought is a global challenge that requires close cooperation between research institutions and industries worldwide. Sharing research findings and germplasm resources on drought resistance will be essential for collectively addressing the threat drought poses to agricultural production.

In conclusion, the future of Solanaceae drought resistance research and development will rely on interdisciplinary collaboration and technological innovation. Only through such efforts can crops be effectively adapted to extreme climate conditions, ensuring global food security in the years to come. In addition, while significant progress has been made in understanding the molecular basis of drought tolerance in Solanaceae crops, the mechanisms underlying drought avoidance and drought escape remain underexplored. Future research should focus on unraveling the genetic and molecular basis of these strategies, particularly in stomatal regulation, root architecture, and flowering time, to provide a more holistic approach to improving drought resilience in Solanaceae crops.

## Figures and Tables

**Table 1:** Transcription factors that regulate drought resistance in Solanaceae.

Species	Transcription factors	Function	References
Tomato	*SlMYB49*	Positive regulators	[20]
	*SlMYB50*	Negative regulators	[4]
	*SlMYB55*	Negative regulators	[21]
	*SlMYB-78-like*	Positive regulators	[22]
	*SlWRKY81*	Negative regulators	[23,24]
	*SlWRKY8*	Positive regulators	[25]
	*SlWRKY6*	Positive regulator	[26]
	*ERF5*	Positive regulators	[27]
	*SlERF84*	Positive regulators	[28]
	*SlEREB1*	Negative regulators	[29]
	*SlNAC4*	Positive regulators	[30]
	*SlNAC35*	Positive regulators	[31]
	*SlNAC042*	Positive regulators	[32]
	*SlNAP1*	Positive regulators	[33]
	*SlNAC2*	Positive regulators	[34]
	*SlNAC6*	Positive regulators	[35]
	*SlAREB1*	Positive regulators	[8]
	*SlbZIP38*	Negative regulators	[36]
	*SlbZIP1*	Positive regulators	[37]
	*HD-Zip*	Positive regulators	[38]
Pepper	*MYB1*	Positive regulators	[39]
	*CaWRKY27*	Negative regulators	[40]
	*CaNAC072*	Negative regulators	[41]
	*CaNAC46*	Positive regulators	[42]
	*CaATBZ1*	Negative regulators	[43]
	*CaDILZ1*	Positive regulators	[15]
	*CaDRHB1*	Positive regulators	[44]
	*CaGRA1*	Positive regulators	[45]
Potato	*StMYB1R-1*	Positive regulators	[46]
	*StWRKY1*	Positive regulators	[47]
	*StWRKY2*	Positive regulators	[48]
	*StWRKY48*	Negative regulators	[49]
	*StNAC2*	Positive regulators	[50]
	*StNAC053*	Positive regulators	[51]

## Data Availability

The authors confirm that there is no data to be made available for this review.
